# Development and validation of method for defining conditions using Chinese electronic medical record

**DOI:** 10.1186/s12911-016-0348-6

**Published:** 2016-08-20

**Authors:** Yuan Xu, Ning Li, Mingshan Lu, Robert P. Myers, Elijah Dixon, Robin Walker, Libo Sun, Xiaofei Zhao, Hude Quan

**Affiliations:** 1 Beijing YouAn Hospital, Capital Medical University, 8 Xitoutiao Fengtai, Beijing, 100069 China; 2 Department of Community Health Sciences, University of Calgary, Calgary, Alberta Canada; 3 Department of Economics, University of Calgary, Calgary, Alberta Canada; 4 Liver Unit, Division of Gastroenterology and Hepatology, Department of Medicine, University of Calgary, Calgary, Alberta Canada; 5 Division of General Surgery, Faculty of Medicine, University of Calgary, Calgary, Alberta Canada

**Keywords:** Extraction method, Validation, Case definition, Electronic medical record, Liver disease

## Abstract

**Background:**

The adoption of the electronic medical record (EMR) is rapidly growing in China. Constantly evolving, Chinese EMRs contain vast amounts of clinical and financial data, providing tremendous potential for research and policy use; however, they are only partially standardized and contain free text or unstructured data. To utilize the information contained in Chinese EMRs, the development of data extraction methodology is urgently needed. The purpose of this study is to develop and validate methods to extract clinical information from the Chinese EMR for research use.

**Methods:**

Using 2010 to 2014 EMR data from YouAn Hospital, a large teaching hospital affiliated with Capital Medical University in Beijing, China, we developed extraction methods including 40 EMR definitions for defining 6 liver disease, 5 disease severity conditions, and 29 comorbidities and treatments. We conducted a chart review of 450 randomly selected EMRs. Using physician chart review results as a reference, sensitivity, specificity, positive predictive value (PPV), and negative predictive value (NPV) were calculated to validate each EMR definition.

**Results:**

The sensitivity of the 6 EMR definitions for liver diseases ranged from 78.9 to 100.0 %, and PPV ranged from 82.1 to 100.0 %. The sensitivity of the 5 definitions on disease severity conditions ranged from 91.0 to 100.0 %, and PPV ranged from 79.2 to 100.0 %. Among the 29 EMR definitions for comorbidities and treatments, 23 had sensitivity over 90.0 % and 25 had PPV over 80.0 %. The specificity and NPV for all 40 EMR definitions were over 90.0 %.

**Conclusion:**

The extraction method developed is a valid way of extracting information on liver diseases, comorbidities and related treatments from YouAn hospital EMRs. Our method should be modified for application to other Chinese EMR systems, following our framework for extracting conditions.

**Electronic supplementary material:**

The online version of this article (doi:10.1186/s12911-016-0348-6) contains supplementary material, which is available to authorized users.

## Background

Epidemiological and clinical outcome studies of liver diseases require large databases and rich case mix information to incorporate severity assessment and risk adjustment in order to generate robust results [1]. Many researchers in China are currently seeking to explore liver disease using clinical data, given that China has a high prevalence of liver diseases. Chronic hepatitis is the major cause of primary liver cancer (PLC) in the Chinese population. In China, the prevalence of hepatitis B virus infection is 7.2 %, and an estimated 20 million people suffer from chronic hepatitis B [2, 3]. The high prevalence of chronic hepatitis has resulted in a high incidence of PLC: an estimated 360,000 new cases of PLC occur in China each year [4].

The implementation of the electronic medical record (EMR) in China has been rapidly growing since 2006 [5]. Developing the EMR has been identified as a primary focus for China’s new and on-going health care reforms [6]. Currently, most hospitals in China use EMR systems. The coverage, functionality, and interoperability of the EMR will continue to be greatly improved as these developments take place. In addition to the advantages of the EMR including large geographical coverage and easy access, they also hold tremendous potential to provide rich and timely longitudinal clinical data for research [5, 7]. Researchers worldwide have recognized the potential value of EMRs and tremendous efforts are underway to advance research in EMR data science [8–10].

The utility of Chinese EMRs for research, however, must take into account the specific features of the EMR systems. Similarly with other countries, EMR systems in China contain longitudinal and comprehensive documentation of clinical care and management of patients’ hospitalizations. Information on diagnoses, laboratory tests, radiology examinations, hospital costs, procedure reports, and prescriptions is recorded. EMR data is inputted by physicians and regularly audited by hospital medical record departments, as the quality of the EMR is part of the physicians’ monthly performance evaluations. In spite of national standards to improve interoperability, however, EMR systems in China are only partially standardized [5–7]. This is because each hospital in China chooses its own vendor to develop an EMR system, with different vendors having developed different systems. These EMR systems incorporate many free-text fields with unstructured data; structured drop-down lists are limited to the documentation of demographics, physical examinations, and drug prescriptions. Additionally, the standardized coding system, such as the International Classification of Diseases (ICD), is not used to document clinical conditions in Chinese EMRs [5–7].

In recent years, methodologies that extract information from EMRs have been improved. One outstanding example is the electronic phenotyping algorithms that were developed by the electronic medical records and genomics (eMERGE) network [11, 12]. These algorithms require the ICD codes and Current Procedural Terminology (CPT) to be present in EMR data [11, 13]. However, in addition to language differences (*i.e.,* English versus Chinese), the lack of standardized coding systems (e.g., ICD) in Chinese EMRs makes existing extraction methods, such as the eMERGE phenotyping algorithms, inapplicable to Chinese EMRs. Another barrier for using Chinese EMRs to define disease conditions is the lack of validated tools for dealing with unstructured free-text. Natural Language Processing (NLP) is a sophisticated tool that is used to convert unstructured free-text into structured data. However, well-established and widely validated NLP techniques for dealing with Chinese EMRs are not currently available. Studies of NLP for Chinese EMRs are limited, with most having been developed for one or several specific sections or fields of the EMR [14–16]. Additionally, clinical expertise is needed to define conditions (such as liver diseases, symptoms and comorbidities) according to practice pathways because the EMRs in China document longitudinal clinical information on patients, but do not document diagnoses in a standardized or systematic way. For example, a patient’s illness history, biomarker level, and imaging reports represent some of the information that is captured in EMRs and can be combined to define PLC.

In order to leverage the use of existing Chinese EMRs, there is a need to develop new data extraction methods that incorporate clinical expertise and make use of free-text and non-standardized coded data to accurately define conditions. In this study, we aimed to develop and validate extraction methods to define disease conditions in Chinese EMRs, specifically focusing on liver diseases, which are widely used in liver disease outcome and risk adjustment studies. Our methodology also provides a framework for constructing and defining other conditions in Chinese EMRs.

## Methods

### Data source

**Fig. 1:**
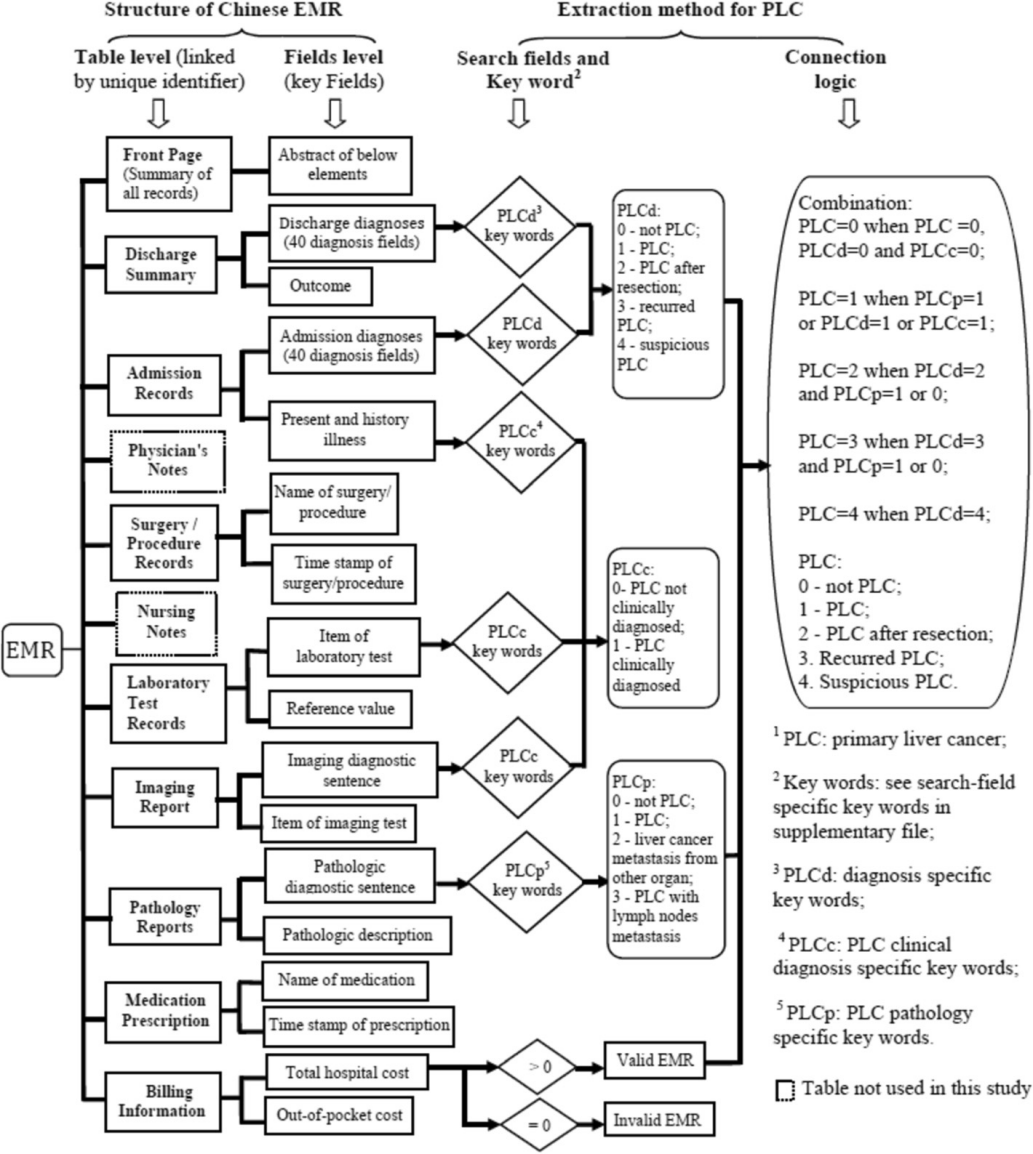
The main structure of Chinese EMR and extraction method for Primary Liver Cancer (PLC)

The EMR data was collected and extracted from the EMR system at YouAn hospital, which is affiliated with Capital Medical University in Beijing, China. YouAn is one of the leading teaching hospitals in China. It specializes in delivering care to patients with liver diseases, and treats over 300,000 patients annually from all regions of China. The EMR system in use at YouAn hospital was developed using Structured Query Language (SQL), a method that is widely used across other Chinese EMR systems. For each patient, the EMR contains several sections, including a front page (completed after discharge) that summarizes the hospitalization, as well as an admission record, discharge record, surgery/procedure record, death record, laboratory test results, radiology test results, pathology reports, physician notes, hospitalization cost records, and electronic drug prescriptions (see Fig. 1, which illustrates the main structure of this EMR). The sections for laboratory test results, electronic prescriptions, and hospitalization cost records are entirely structured, without any free text. However, the front page, admission record, discharge record, and radiology test results are only semi-structured and contain both structured drop-down lists and free-text fields. YouAn assigns a unique identification number to each patient, which allows for the linkage of multiple EMR sections.

For this study, we included patients who were admitted to YouAn hospital between January 1, 2010 and August 15, 2014. This resulted in a sample of 85,524 EMRs. All the included patients consented to having their EMRs used for scientific research, as the consent form was integrated directly into the EMR. It is estimated that over 95 % of patients admitted to YouAn hospital have agreed and signed this consent form.

### EMR extraction method

We developed 40 EMR definitions for 6 liver diseases, 5 disease severity conditions and 29 comorbidities and treatments (Additional file 1: Table S1, which shows a list of EMR definitions for liver diseases, disease severity conditions, comorbidities and treatments developed in this study). We queried the EMR data using the SQL system. There were three steps in the development of each EMR definition. First, we developed the keywords that were corresponding to different free-text entries of the EMR. Keywords included Chinese words and punctuation, and were combined using “AND”/“OR” operators. Because there are various ways of indicating a clinical condition in free text in Chinese, we attempted to include all potential disease descriptions and specific terms in a group of search terms that are specific to each condition based on our knowledge of physician documentation style for a specific disease and the guidelines or rules for documenting clinical information in a particular EMR section. Second, we selected corresponding sections and fields in the EMR for searching the keywords, such as the 40 diagnosis fields in the admission record, conclusive sentences in pathology or radiology reports, and laboratory results. We determined the areas of focus by considering the disease diagnostic pathway, as well as the availability, completeness, and temporality of information in the EMR. The third step consisted of logically combining the search results from selected fields. Our logical combination embedded the inclusion and exclusion criteria in light of clinical expertise. We defined the liver diseases using national and international practice guidelines [17–21]. An example of our extraction method for PLC is illustrated in Fig. 1. Finally, we tested the EMR definitions on a 10 % sample of the reviewed EMRs (45 EMRs) to include comprehensive lists of the keywords and detect and fix errors, such as inadequate inclusion of search fields, misspelling, wrong search sections, and errors in SQL syntax and clinic logics. Through an iterative process of testing and revising, we finalized the 40 EMR definition algorithms (available at: http://themethodshub/toolbox/downloads).

In our method, the common liver diseases extracted from the EMR included: PLC, hepatitis B, hepatitis C, fatty liver, alcoholic liver diseases and cirrhosis. In addition, the following disease severity conditions were also extracted: hepatic encephalopathy, spontaneous bacterial peritonitis, variceal hemorrhage, hepatorenal syndrome, and ascites. Along with other laboratory test results (*i.e.*, prothrombin time and serous level of albumin, creatinine, sodium and total bilirubin), these variables can be used to calculate a Child-Pugh score [22], model for end-stage liver disease (MELD) index [23], MELD-Sodium index (MELD-Na) [24] and five-variable MELD (5vMELD) index [24]. These indexes are widely used to assess the severity of liver disease in clinical settings. For the purpose of risk adjustment, we extracted the comorbidities included in the Charlson Comorbidity Index (CCI) [25] and Elixhauser Comorbidity Index (ECI) [26], which are commonly used risk adjustors. We extracted treatment or procedure measures, which are highly recommended for consideration in outcome and risk adjustment studies [27].

### EMR review

Using patients’ unique identification number, we randomly selected 450 patients and extracted their EMRs (450 EMRs) from the entire 85,524 EMRs. To validate the EMR definitions, two hepatologists who were blinded to the EMR definitions reviewed the EMRs. For training purposes, each physician first reviewed the same 20 EMRs independently. Study team members ensured the accuracy and consistency of the EMR review process by comparing physician review results. Inconsistencies or uncertainties in decisions were brought to the team for discussion. Each physician then reviewed 250 full EMRs, of which 50 EMRs were independently reviewed by both reviewers to assess the inter-rater agreement. *Kappa*-statistic (*K*-statistic) was calculated to assess inter-rater agreement between physician reviewers. Of the 40 EMR definitions, we were able to calculate a *k* statistic for 32 definitions. Of the 32 definitions, 30 definitions had a *k*-statistic between 0.4 and 1.0, and 2 definitions were between 0.2 and 0.4. It was not possible to calculate a *k*-statistic for the remaining 8 definitions, given that there were “0” cells in the contingency tables due to the low prevalence of some conditions. In this case, the observed agreement was used to evaluate the agreement (over 85.0 %).

### Statistical analysis

Descriptive statistics were calculated for demographic characteristics, liver diseases, disease severity conditions, and comorbidities. Sensitivity, specificity, positive predictive value (PPV), and negative predictive value (NPV) and its 95 % confidence intervals of the EMR definitions were assessed based on the reference of EMR review results.

We tried several different EMR definitions for each individual disease, symptom or treatment. Three types of EMR definitions were constructed: 1) definition based on diagnosis information (admission and discharge) only; 2) definition based on supporting clinical evidence only, such as the information from the laboratory test results, imaging results, pathologic reports, illness history, procedure records and prescription; and, 3) definition based on both diagnosis and supporting clinical evidence. For each disease, symptom or treatment, after comparing the validity of different EMR definitions, the one with highest validity was chosen.

All statistical analyses were performed using SAS v. 9.4 (SAS Institute, Cary, NC) and Stata v. 12.0 (StataCorp, College Station, TX).

## Results

Among the 450 randomly selected patients whose records were reviewed for validation, 234 (52.0 %) were male and 328 (72.9 %) were between 18 and 64 years old (Table 1). The prevalence of liver diseases in the sample was 21.3 % for PLC, 32.7 % for cirrhosis, 45.1 % for hepatitis B, 8.9 % for hepatitis C, 7.1 % for alcoholic liver disease, and 4.0 % for fatty liver. Of the sample, 68.9 % had at least one of the comorbidities included in CCI and 71.8 % had at least one of the comorbidities included in ECI.

**Table 1:** Patient’s Characteristics (*N* = 450)

Characteristic	Percent, % (n)
Male	52.0 (234)
Age (year)	
<18	17.6 (79)
18-64	72.9 (328)
>64	9.6 (43)
Primary liver cancer	21.3 (96)
Cirrhosis	32.7 (147)
Hepatitis B	45.1 (203)
Hepatitis C	8.9 (40)
Fatty liver	4.0 (18)
Alcoholic liver disease	7.1 (32)
Spontaneous bacterial peritonitis	14.9 (67)
Variceal hemorrhage	4.4 (20)
Hepatorenal syndrome	1.1 (5)
Hepatic encephalopathy	8.9 (40)
Ascites	27.6 (124)
Number of Charlson comorbidities	
0	31.1 (140)
1	33.3 (150)
2	22.7 (102)
3 and more	12.9 (58)
Number of Elixhauser comorbidities	
0	28.2 (127)
1	24.4 (110)
2	15.1 (68)
3 and more	32.2 (145)

We validated 40 EMR definitions, which were classified into three groups: 6 definitions for liver diseases, 5 definitions for severity of liver disease, and 29 definitions for comorbidities and treatments. The validity of 11 EMR definitions for liver diseases and disease severity conditions is shown in Table 2. For the 6 definitions for liver diseases (*i.e.,* PLC, hepatitis B, hepatitis C, cirrhosis, fatty liver, and alcoholic liver diseases), the sensitivity ranged from 78.9 to 100.0 %, and PPV ranged from 82.1 to 100.0 %. The sensitivity of 5 definitions for the severity of liver disease (*i.e.,* HE, ascites, SBP, VH and HR) ranged from 91.0 to 100.0 % (see Table 2). The specificity and NPV for all 40 EMR definitions were over 90.0 %.

**Table 2:** Validity of EMR^a^ definitions for liver disease

Variable	Sensitivity % (95 % CI^b^)	Specificity % (95 % CI)	PPV^c^ % (95 % CI)	NPV^d^ % (95 % CI)
Liver disease				
Primary liver cancer	100.0 (96.2, 100.0)	98.9 (97.1, 99.7)	96.0 (90.1, 98.9)	100.0 (99.0, 100.0)
Hepatitis B	88.2 (82.9, 92.3)	98.8 (96.5, 99.8)	98.4 (95.3, 99.7)	91.0 (87.0, 94.2)
Hepatitis C	82.5 (67.2, 92.7)	99.5 (98.3, 99.9)	94.3 (80.8, 99.3)	98.3 (96.6, 99.3)
Fatty liver	100.0 (81.5, 100.0)	100.0 (99.2, 100.0)	100.0 (81.5, 100.0)	100.0 (99.2, 100.0)
Alcoholic liver disease	100.0 (89.1, 100.0)	98.3 (96.6, 99.3)	82.1 (66.5, 92.5)	100.0 (99.1, 100.0)
Cirrhosis	78.9 (71.4, 85.2)	98.4 (96.2, 99.5)	95.9 (90.6, 98.6)	90.6 (86.9, 93.5)
Liver disease severity				
Spontaneous bacterial peritonitis	91.0 (81.5, 96.6)	99.7 (98.6, 100.0)	98.4 (91.3, 100.0)	98.5 (96.7, 99.4)
Variceal hemorrhage	95.0 (75.1, 99.9)	98.8 (97.3, 99.6)	79.2 (57.9, 92.9)	99.8 (98.7, 100.0)
Hepatorenal syndrome	100.0 (47.8, 100.0)	100.0 (99.2, 100.0)	100.0 (47.8, 100.0)	100.0 (99.2, 100.0)
Hepatic encephalopathy	100.0 (91.2, 100.0)	100.0 (99.1, 100.0)	100.0 (91.2, 100.0)	100.0 (99.1, 100.0)
Ascites	95.2 (89.8, 98.2)	99.1 (97.3, 99.8)	97.5 (92.9, 99.5)	98.2 (96.1, 99.3)

^a^ *EMR* electronic medical records; ^b^ *CI* confidence interval; ^c^ *PPV* positive predictive value; ^d^ *NPV* negative predictive value

The summary of the validity for the additional 29 EMR definitions is shown in Table 3 (for detailed validity results of these 29 EMR definitions for comorbidities and treatments see Additional file 1: Table S2). The sensitivity of the 29 EMR definitions ranged from 64.7 to 100.0 %; 23 definitions had sensitivity over 90 %. The PPV of the 29 EMR definitions ranged from 64.1 to 100 %; 25 definitions had a PPV of over 80.0 %.

**Table 3:** Number and percent of EMR^a^ definitions for comorbidities or treatments by validity range out of 29 EMR definitions

Range	Sensitivity	Specificity	PPV^b^	NPV^c^
60.0 % - 69.9 %	3 (10.3 %)	0	2 (6.9 %)	0
70.0 % - 79.9 %	3 (10.3 %)	0	2 (6.9 %)	0
80.0 % - 89.9 %	0	0	5 (17.2 %)	0
≥90.0 %	23 (79.3 %)	29 (100 %)	20 (69.0 %)	29 (100 %)

^a^ *EMR* electronic medical record; ^b^ *PPV* positive predictive value; ^c^ *NPV* negative predictive value

## Discussion

The current rapid development of EMRs in China presents great potential for health researchers and policy makers. However, significant challenges remain on how to make use of the information present in Chinese EMRs, as their special features make the application of existing data extraction methods infeasible. In this study, we developed an EMR data extraction method including 40 EMR definitions for liver diseases, liver disease severity conditions, comorbidities and treatments using Chinese EMRs. The validity of the EMR definitions developed in this study was overall shown to be high, with some variations across definitions. Most of the EMR definitions had over 80.0 % sensitivity (33 out of 40 variables), 90.0 % specificity (all 40 variables), 80.0 % PPV (35 out of 40 variables), and 90.0 % NPV (all 40 variables).

### Validity of the EMR definitions and comparison with other studies

We did not find any other studies that used methods comparable to ours, however to assess the level of validity in our study, we referenced several relevant previous studies. Compared with the case definition of hepatocellular carcinoma (HCC) using pathologic reports and administrative data in the United States [8], our EMR definition for PLC (mainly consists of HCC) had a slightly higher validity with a sensitivity of 100.0 % versus 96.0 %, and a specificity of 98.9 % versus 97.0 %. One study that validated ICD administrative data at the University of Pennsylvania Health System [28] found a lower PPV than ours for hepatitis C (88.0 % versus 94.3 %) and hepatitis B (81.3 % versus 98.4 %). Our EMR definitions for comorbidities such as hypertension, diabetes, chronic obstructive pulmonary disease, and depression also had higher validity than another study that used Canadian primary healthcare EMRs [9]. In addition, the EMR definition for acquired immune deficiency syndrome (AIDS) also reported very high validity, similar to the findings of another study [29]. The high validity of our definitions is likely related to the high quality of physician documentation, as well as the structure of Chinese EMRs. In China, there are specific criteria/rules for physicians to follow when documenting clinical information using EMRs (e.g., one diagnosis field assigned for each diagnosis). The EMRs are also fully structured for reporting laboratory tests (i.e., serous level of albumin, creatinine, sodium and total bilirubin, and prothrombin time).

Our EMR definitions achieved PPV results similar to those of the eMERGE phenotype algorithms. Among different sites of eMERGE, PPV ranged from 98.2 to 100.0 % for diabetes, 84.0 to 100.0 % for hypertension, 73.0 to 84.0 % for dementia, and 92.0 to 96.0 % for chronic kidney disease [30]. An EMR definition of myocardial infarction using the United Kingdom’s electronic clinical data [31] had a higher PPV than ours (85.3 % versus 66.7 %). Given the fact that PPV is prevalence sensitive, the lower PPV of the myocardial infarction definition in our study might be partially explained by the low prevalence (0.44 %) of this condition in our data set.

Notably, the validity of the developed EMR definitions varied by extracted diseases/treatments, with low sensitivity for cirrhosis (78.9 %), fluid and electrolyte disorders (65.4 %), valvular disease (66.7 %), metastatic solid tumor (72.7 %) and peptic ulcer disease (64.7 %). There are several possible explanations. First, the diagnoses of these conditions are complex and/or contain a wide range of sub-diagnoses, such as metastatic solid tumor and peptic ulcer disease, which requires the integration of clinical evidence and physician judgment. Second, disease-related information is not always well documented in EMRs (e.g., a pathology report is only available for patient who underwent tissue biopsy or surgery). Third, some minor diseases, such as fluid and electrolyte disorders, have a relatively short recovery time, and physicians may not document the diagnosis during hospitalization.

Our results indicated that the validity of some EMR definitions only slightly improved after adding laboratory test results or prescription information. For example, after adding prescription information into the EMR definition for hepatitis B, which previously only contained diagnosis information, sensitivity improved slightly from 86.8 to 88.2 % and PPV improved from 97.8 to 98.4 %. We found similar results for hepatic encephalopathy and AIDS. This indicates that the diagnoses in EMRs may be sensitive enough for developing case definitions for conditions, such as hepatitis B, hepatic encephalopathy, and AIDS.

### Application of the developed extraction method

The developed EMR definition consists of three components: Chinese language search terms, targeted fields to search, and the logical connection of the extracted information. The search terms can be used directly in other EMRs, given that we developed them based on the physician documentation style used for a specific disease. Although the specific details of EMR systems vary across hospitals in China, the high-level structure and data fields used in this study remain similar under national EMR standards [6]. Chinese EMR systems contain all necessary and essential sections or fields facilitating the application of our extraction method. The third component was developed based on the clinical practice pathway or diagnostic guidelines for each disease. However, our extracting method should be modified to fit unique Chinese EMR systems. For extracting variables not included in this study, the framework for constructing the extraction method includes: 1) determining the search fields; in most scenarios, EMRs include diagnoses in admission or discharge records, conclusive sentences in pathology or imaging reports, and corresponding laboratory test results; 2) developing keywords for searching the fields that are specific to the disease, based on the clinical expertise and documentation styles used by physicians; and 3) combining the search results based on the specific diagnostic guidelines.

Our extraction method utilized the free-text within the Chinese EMR, which contains rich clinical information and accounts for a large part of the EMR. In existing EMR extraction methods, NLP is commonly used to work with free text. We did not use NLP because well-established and widely validated NLP for dealing with various Chinese EMRs is currently not available. Instead, our extracting method searched for documented keywords. This technique was practical and feasible because not only do certain sections of the Chinese EMR with free text data entries consist of one conclusive sentence or several words, our methods also searched a small number of the most relevant free-text entries (diagnoses and conclusive sentences). Generally, in Chinese EMRs, these free-text entry fields are generic in terms of what diagnosis or other information can be entered, therefore the variation in how these fields are recorded by physicians is limited, as physicians are required to follow standardized criteria when inputting diagnoses or conclusive sentences in these fields. This enhances the potential of our methodology to be applied to other Chinese EMRs.

EMRs serve as a tool to facilitate medical documentation in the clinical setting, and also provide rich data to contribute to evidence-based medicine. The new methodology proposed in this study for extracting information from the Chinese EMRs could transfer rich EMR data into a ready-for-analysis format, and provide timely information for prognosis prediction, risk adjustment, and quality assessment. Our methods will enable and leverage clinical and epidemiological applied studies to use EMR data for generating knowledge for evidence-based medical practice.

### Challenges and limitations

We faced challenges common to the development of all EMR data extraction methods. This included how to best handle the combination of information derived from different parts of the EMR, and the temporality of information, for instance blood test results, missing values, and free text components.

Our study has several limitations. First, we used an EMR physician review as our gold standard, although the validity of reviewed EMRs is unknown. However, it is widely agreed upon that performing a medical record review is an adequate standard for use in validation studies [32]. This may be especially true for Chinese hospitals in which EMRs are routinely audited by medical record departments. The quality of the documentation is considered an indicator of physician performance in monthly evaluations. Second, aside from the reported 40 EMR definitions, we developed an additional 10 EMR definitions for other liver diseases and comorbidities (i.e., intrahepatic cholangiocarcinoma, pulmonary circulation disorders, paralysis, lymphoma, coagulopathy, psychoses, congestive heart failure, peripheral vascular disease, weight loss and dementia) (see Additional file 1: Table S1). However, we were unable to assess the validity of these conditions due to low prevalence. Third, we developed and validated EMR definitions in one hospital that specializes in diagnosing and treating liver disease and therefore the statistics of validity were influenced by disease prevalence in our sample. To apply our methods, users should modify our data extraction methodology to fit their EMR systems and further validate this in their EMRs.

## Conclusion

The developed EMR definitions presented in this study had high validity. Our method should be modified for application to other Chinese EMR systems, following our framework for extracting conditions. Our method contributes to current efforts that attempt to make use of the robust clinical data sources in China to leverage outcome researches. Future research should focus on testing our extraction method in other EMR data and modifying it for use with other conditions. This will in turn provide informative, timely and interoperable clinical data for researchers and policy makers.

## Abbreviations

5vMELD, five-variable MELD; AIDS, acquired immune deficiency syndrome; CCI, Charlson comorbidity index; CPT, current procedural terminology; ECI, Elixhauser comorbidity index; eMERGE, electronic medical records and genomics; EMR, electronic medical record; HCC, hepatocellular carcinoma; ICD-9-CM, International classification of disease – 9th version - clinical modification; MELD, model for end-stage liver disease; MELD-Na, MELD-Sodium; NLP, natural language processing; NPV, negative predictive value; PLC, primary liver cancer; PPV, positive predictive value; SQL, structured query language

## Additional file

Additional file 1: Table S1. EMR definitions for liver diseases, disease severity conditions, comorbidities and treatments. **Table S2.** Validity results of the 29 EMR definitions for comorbidities and treatments. (DOC 251 kb)
