# Supplementary material for: Development and validation of method for defining conditions using Chinese electronic medical record
Source: BMC Med Inform Decis Mak. 2016 Aug 20;16:110. doi: 10.1186/s12911-016-0348-6 (PMC4992264; doi:10.1186/s12911-016-0348-6)
Supplement: Additional file 1: Table S1. — EMR definitions for liver diseases, disease severity conditions, comorbidities and treatments. Table S2. Validity results of the 29 EMR definitions for comorbidities and treatments. (DOC 251 kb) [file 12911_2016_348_MOESM1_ESM.doc]

Additional file of **"Development and Validation of Method for Defining Conditions Using Chinese Electronic Medical Records"**

**Table 1 EMR definitions for liver diseases, disease severity conditions, comorbidities and treatments**

| **Variable and Connection Logic** | **Search Term** |
| --- | --- |
| **LIVER DISEASE** |  |
| **Primary Liver Cancer (PLC):** |  |
| PLCp (PLC pathology)  PLCp = 0 if none of following;  PLCp = 1 when Term I are found or PLC=3 & Term IV are found in Pathology Report;  PLCp =2 when PLCp=1 and Term II are found in Pathology Report;  PLCp =3 when PLCp =2 and Term III are found in Pathology Report.  (PLCp: 0 - not PLC; 1 - PLC; 2 - liver cancer metastasis from other organ; 3 - PLC with lymph nodes metastasis). | PLCp:  I. "肝?癌" or "肝?恶性" or "肝?肉瘤"  II. "肝转移" or "肝门部胆管?" or "转移癌" or "肝血管内皮瘤" or "肝血管平滑肌脂肪瘤"  III. "淋巴结转移"  IV. "淋巴结未见转移" |
| PLCd (PLC diagnosis)  PLCd = 0 if none of following;  PLCd = 1 when Term I are found in either Admission or Discharge Diagnoses;  PLCd = 2 when Term I and Term II are found in either Admission or Discharge Diagnoses;  PLCd = 3 when Term II and Term III are found in either Admission or Discharge Diagnoses;  PLCd = 4 when Term IV are found in either Admission or Discharge Diagnoses;  (PLCd = 0 - not PLC; 1 - PLC; 2 - PLC after resection; 3- recurred PLC; 4- suspicious PLC). | PLCd:  I. ("肝" & "癌") or ("肝" & "恶性")  II. "术后"  III. "术后复发"  IV. ("肝" & "占位") or ("肝" & "结节") or ("肝" & "肿物") or "肝癌待排" or "肝癌可能性大" |
| PLCc (PLC clinical diagnosis)  PLCc = 0 if not = 1;  PLCc = 1 when (Term II are found but Term I is not found in Illness History or when Term III are found in Admission or Discharge Diagnoses) & (Term IV are found in CT or MRI results) and (serous AFP more than 400 ng/ml)  (PLCc: 0- not clinically diagnosed PLC; 1 - clinically diagnosed PLC ) | PLCc:  I. "否认?肝" or "无?肝"  II. "乙肝" or "慢?乙?肝" or "丙肝" or "肝?病" or "酒?肝"  III. "慢?肝" or "?乙肝" or "?丙肝" or "肝硬化" or "HBV" or "HCV"  IV. ("肝" & "占位") or ("肝" & "结节") or ("肝" & "肿物") or "肝癌可能性大" |
| Combination:  PLC = 0 when PLCp = 0, PLCd = 0 and PLCc =0;  PLC = 1 when PLCp = 1 or PLCd = 1 or PLCc = 1;  PLC = 2 when PLCd = 2 and PLCp = 1 or 0;  PLC = 3 when PLCd = 3 and PLCp = 1 or 0;  PLC = 4 when PLCd = 4;  (PLC = 0 - not PLC; 1 - PLC; 2 - PLC after resection; 3. recurred PLC; 4. suspicious PLC). | |
| **Hepatocellular Carcinoma_ pathology (HCCp):** |  |
| HCCp = 0 if not 1;  HCCp = 1 when search terms are found in Pathology Report.  (HCCp: 0 - no HCC diagnosed by pathologist; 1 - HCC diagnosed by pathologist) | "肝细胞?癌" |
| **Intrahepatic Cholangiocarcinoma _pathology (ICCp):** |  |
| ICCp = 0 if not 1;  ICCp = 1 when search terms are found in Pathology Report.  (ICCp: 0 - no ICC diagnosed by pathologist; 1 - ICC diagnosed by pathologist) | ("癌" & "肝内胆管") or ("腺癌"&"肝"&"胆管") or ("恶性" & "肝内胆管") |
| **Hepatitis B (HB):** |  |
| HBp (HB pathology):  HBp = 0 if none of following;  HBp = 1 when search terms are found in Pathology Report.  (HBp: 0 - no HB diagnosed by pathologist, 1 - HB diagnosed by pathologist ) | HBp:  "乙肝" or "肝炎乙型" or "乙型肝炎" or "肝硬化?乙型" |
| HBa (Hepatitis B virus & antigen):  Searching the name of the item of blood test  HBa = 0 if none of following;  HBa = 1 when the result of Term I is " + " and the result of Term II is " + " and the result of Term III is " - " . | HBa:  I. "*乙型肝炎表面抗原" or "乙型肝炎表面抗原滴度"  II. "*乙型肝炎核心抗体" or "乙型肝炎核心抗体（快速）" or "乙型肝炎核心抗体IgM"  III. "*乙型肝炎表面抗体" or "乙型肝炎表面抗体" |
| HBn (Hepatitis B virus DNA):  Searching the name of the item of blood test  HBn = 0 when the result of Term I < 500 copies/ml  HBn = 1 when the result of Term I > 500 copies/ml  (HBn: 0 - Hepatitis B virus DNA negative, 1 - Hepatitis B virus DNA positive) | HBn:  "荧光定量乙肝病毒脱氧核糖核酸" or "荧光定量乙肝病毒脱氧核糖核酸'" |
| HBd (HB admission/discharge diagnosis):  HBd = 0 if not 1;  HBd = 1 when search terms are found in admission/discharge diagnoses.  (HBd: 0 - no HB diagnosed, 1 - HB diagnosed ) | HBd:  "乙肝" or ("肝" & "乙型") or "HBV" |
| HBm (Hepatitis B virus western medication):  HBm = 0 if not 1;  HBm = 1 when search terms are found in prescriptions.  (HBVm: 0 - no anti-Hepatitis B virus western medication used, 1 - anti-Hepatitis B virus western medication used) | HBm:  "干扰素-α" or "阿德福韦" or "恩替卡韦" or "拉米夫定" or "替比夫定" or "利巴韦林" |
| Combination:  HB = 0 when HBp = 0, HBa = 0, HBn = 0, HBd = 0 and HBm = 0;  HB = 1 when HBp = 1 or HBa = 1 or HBn = 1 or HBd = 1 or HBm = 1.  (HB: 0 - not Hepatitis B; 1 - Hepatitis B) | |
| **Hepatitis C (HC):** |  |
| HCp (HC pathology)  HCp = 0 if not = 1;  HCp = 1 when search terms are found in Pathology Report.  (HCp: 0 - no HC diagnosed by pathologist, 1 - HC diagnosed by pathologist ) | HCp:  "丙型" or "丙肝" |
| HCa (Hepatitis C virus antibody & antigen)  Searching the name of the item of blood test  HCa = 0 if not = 1;  HCa = 1 when the result of search term is " + ".  (HCa: 0 - Hepatitis C virus antibody and antigen negative, 1 - Hepatitis C virus antibody and antigen positive) | HCa:  "丙型肝炎抗体" |
| HCr (Hepatitis C virus RNA):  Searching the name of the item of blood test  HCr = 0 when the result of search term < 1000 copies/ml  HCr = 1 when the result of search term > 1000 copies/ml  (HCr: 0 - Hepatitis C virus RNA negative, 1 - Hepatitis C virus RNA positive) | HCr:  "荧光定量丙肝病毒脱氧核糖核酸" or "丙型肝炎病毒(HCV)RNA测定" |
| HCd (HC admission diagnosis):  HCd (HC admission/discharge diagnosis):  HCd = 0 if not 1;  HCd = 1 when search terms are found in admission/discharge diagnoses.  (HCd: 0 - no HC diagnosed, 1 - HC diagnosed) | HCd:  "丙肝" or "丙型" or "HCV" |
| Combination:  HC = 0 when HCp = 0, HCa = 0, HCn = 0, HCd = 0 and HCm = 0;  HC = 1 when HCp = 1 or HCa = 1 or HCn = 1 or HCd = 1 or HCm = 1.  (HC: 0 - not Hepatitis C; 1 - Hepatitis C) | |
| **Fatty Liver (FL):** |  |
| FLp (Fatty Liver Pathology):  Searching Pathology Report  FLp= 0 if not = 1;  FLp = 1 when Term I are found first and then Term II are not found, or when Term I are found first and then Term II are not found, sequentially Term III are found.  (FLp: 0 - fatty liver not diagnosed by pathologist, 1 - fatty liver diagnosed pathologist) | FLp:  I. "脂肪"  II. "酒精性脂肪" or "肛周" or "肠周" or "腮腺" or "血管脂肪" or "成熟脂肪" or "脂肪瘤"  III. "非酒精性脂肪" |
| FLd (Fatty liver admission/discharge diagnosis):  Searching Admission or Discharge Diagnoses  FLd = 0 when not = 1;  FLd = 1 when Term I are found,  (FLp: 0 - fatty liver not diagnosed, 1 - fatty liver diagnosed) | FLd:  "非酒精性脂肪" or "脂肪肝" or "脂肪性肝炎" or "单纯性脂肪" or "单纯脂肪" |
| Combination:  FL = 0 when FLp = 0 and FLd = 0;  FL = 1 when FLp = 1 or FLd = 1,  (FL: 0 - not fatty liver, 1 - fatty liver) | |
| **Alcoholic (Al):** |  |
| Alp (Alcoholic liver disease pathology)  Searching Liver Pathology Report  Alp = 0 if not = 1;  Alp = 1 when Term I are found.  (Alp: 0 - alcoholic liver disease not diagnosed by pathologist, 1 - alcoholic liver disease diagnosed by pathologist) | Alp:  I. "酒精" |
| Ald (alcoholic liver disease admission/discharge diagnosis):  Searching Admission or Discharge Diagnoses  Alp = 0 if not = 1;  Alp = 1 when Term I are found first but Term II are not found.  (Alp: 0 - alcoholic liver disease not diagnosed, 1 - alcoholic liver disease diagnosed) | Ald:  I. "酒精"  II. "非酒精" or "无水酒精" or "酒精化学" or "使用酒精" |
| Combination:  Al = 0 when Alp = 0 and Ald = 0;  Al = 1 when Alp = 1 or Ald = 1,  (Al: 0 - not alcoholic liver disease, 1 - alcoholic liver disease) | |
| **Cirrhosis (Cir):** |  |
| Cirp (Cirrhosis pathology):  Searching Pathology Report  Cirp = 0 if not = 1;  Cirp = 1 when Term I are found.  (Cirpa: 0 - cirrhosis not diagnosed by pathologist, 1 - cirrhosis diagnosed by pathologist) | Cirp:  I. "肝?硬化" or "肝？纤维化" |
| Cird (Cirrhosis admission/discharge diagnosis):  Searching Admission or Discharge Diagnoses  Cird = 0 if not = 1;  Cird = 1 when Term I are found first but Term II are not found.  (Cird: 0 - alcoholic liver disease not diagnosed, 1 - alcoholic liver disease diagnosed) | Cird:  I. "肝?硬化" or "肝？纤维化" |
| Combination:  Cir = 0 when Cirp = 0 and Cird = 0;  Cir = 1 when Cirp = 1 or Cird = 1,  (Cir: 0 - not cirrhosis, 1 - cirrhosis) | |
| **LIVER DISEASE SEVERITY** | |
| **Ascites (Asc):** |  |
| Ascad (Ascites at admission)  Searching Admission Diagnoses or results of Ultrasound-B or computerized tomography or magnetic resonance imaging at admission.  Ascad = 0 if not following;  Ascad = 1 when Term I and II are found;  Ascad = 2 when Term I and III are found;  Ascad = 3 when Term I and IV are found;  Ascad = 4 when Term I are found.  (Ascad: 0 - ascites not diagnosed at admission; 1 - slight/mild ascites; 2 - medium ascites; 3 - severe ascites; 4 - ascites with unknown severity) | I. "腹水" or "腹腔积液" or "腹积水";  II. "轻度" or "少量" or "微量" or "极少量";  III. "中度" or "中量";  IV. "重度" or "大量" or "极大量" |
| Ascdis (Ascites at discharge)  Searching Discharge Diagnoses or results of ultrasound-B or computerized tomography or magnetic resonance imaging at discharge.  Ascdis = 0 if not following;  Ascdis = 1 when Term I and II are found;  Ascdis = 2 when Term I and III are found;  Ascdis = 3 when Term I and IV are found;  Ascdis = 4 when Term I are found.  (Ascdis: 0 - ascites not diagnosed at discharge; 1 - slight/mild ascites; 2 - medium ascites; 3 - severe ascites; 4 - ascites with unknown severity) |  |
| **Hepatic Encephalopathy (HE):** |  |
| HEad(HE diagnosed at admission)  Searching Admission Diagnoses and physical examination at admission  HEad = 0 if not following;  HEad = 1 when Term I and II are found;  HEad = 2 when Term I and III are found;  HEad = 3 when Term I and IV are found;  HEad = 5 when Term I and V are found;  HEad = 5 when Term I are found.  (HEad: 0 - HE not diagnosed at admission; 1 - slight; HE; 2 - mild; 3 - medium HE; 4 - severe HE; 5 - HE with unknown severity)  HEdis (HE diagnosed at discharge)  Searching Discharge Diagnoses  HEdis = 0 if not following;  HEdis = 1 when Term I and II are found;  HEdis = 2 when Term I and III are found;  HEdis = 3 when Term I and IV are found;  HEdis = 5 when Term I and V are found;  HEdis = 5 when Term I are found.  (HEdis: 0 - HE not diagnosed at discharge; 1 - slight; HE; 2 - mild; 3 - medium HE; 4 - severe HE; 5 - HE with unknown severity) | I. "肝性脑" or "肝颤" or "扑翼样?颤"  II. "1?" or "Ι?" or "一?" or "前驱期"  III. "2?" or "11?" or "Ⅱ?" or "2-3?" or "二?" or "昏迷前期" or "ΙΙ?"  IV. "111?" or "Ⅲ?" or "三?" or "3?" or "昏睡期" or "ΙΙΙ?"  V. "4?" or "四?" or "IV?" or "昏迷期" or "ΙΙΙΙ度" or "ΙΙΙΙ?" or "1111?" |
| **Hepatorenal syndrome (Hr)：** |  |
| Hrad (Hr diagnosed at admission)  Hrad = 0 if not = 1;  Hrad = 1 when search terms are found in admission diagnoses.  (Hrad: 0 - Hr not diagnosed at admission; 1 - Hr diagnosed at admission)  Hrdis (Hr diagnosed at discharge)  Hrdis = 0 if not = 1;  Hrdis = 1 when search terms are found in discharge diagnoses.  (Hrdis: 0 - Hr not diagnosed at discharge; 1 - Hr diagnosed at discharge) | "肝肾综合" or "肝肾功" |
| **Variceal hemorrhage (Vh)：** |  |
| Vhad (Vh diagnosed at admission)  Vhad = 0 if not = 1;  Vhad = 1 when search terms are found in admission diagnoses.  (Vhad: 0 - Vh not diagnosed at admission; 1 - Vh diagnosed at admission)  Vhdis (Vh diagnosed at discharge)  Vhdis = 0 if not = 1;  Vhdis = 1 when search terms are found in discharge diagnoses.  (Vhdis: 0 - Vh not diagnosed at discharge; 1 - Vh diagnosed at discharge) | "消化道出血" or "食?胃底静脉破裂" or "食?静脉破裂" or "胃底静脉破裂" or "食?胃底曲张静脉破裂" or "食?曲张静脉破裂" or "食?静脉曲张破裂" or "胃底静脉曲张破裂" |
| **Spontaneous Bacterial Peritonitis (SBP)：** |  |
| SBPad (SBP diagnosed at admission)  SBPad = 0 if not = 1;  SBPad = 1 when Term I are found and Term II is not found in admission diagnoses.  (SBPad: 0 - SBP not diagnosed at admission; 1 - SBP diagnosed at admission)  SBPdis (SBP diagnosed at discharge)  SBPdis = 0 if not = 1;  SBPdis = 1 when Term I are found and Term II is not found in discharge diagnoses.  (SBPdis: 0 - SBP not diagnosed at discharge; 1 - SBP diagnosed at discharge) | I. "腹腔感染"  II. "血性" or "耐药" |
| **CHARLSON COMORBIDITIES** | |
| **Myocardial Infarction^1^ (MI)：** |  |
| MId (MI diagnosed at admission/discharge)  MId = 0 if not = 1;  MId = 1 when Term I are found in admission or discharge diagnoses;  MId = 2 when Term II are found in admission or discharge diagnoses..  (MId : 0 - MI not diagnosed, 1 - MI diagnosed, 2 - old MI) | I. "心梗" or "心肌梗"  II. "陈旧性心" or "陈旧心" |
| **Congestive Heart Failure (CHF)：** |  |
| CHFd (CHF diagnosed at admission/discharge)  CHFd = 0 if not = 1;  CHFd = 1 when Term are found in admission or discharge diagnoses.  (CHFd : 0 - CHF not diagnosed, 1 - CHF diagnosed) | "心衰" or "心力衰" |
| **Peripheral Vascular Disease (PVD)：** |  |
| PVDd (PVD diagnosed at admission/discharge)  PVDd = 0 if not = 1;  PVDd = 1 when Term are found in admission or discharge diagnoses.  (PVDd : 0 - PVD not diagnosed, 1 - PVD diagnosed) | "下肢动脉阻塞" or "外周血管性" or "PVD" or "PAD" or "下肢动脉硬化" or "动脉硬化性闭塞" |
| **Cerebrovascular Disease (CEVD)：** |  |
| CEVDd (CEVD diagnosed at admission/discharge)  CEVDd = 0 if not = 1;  CEVDd = 1 when Term are found in admission or discharge diagnoses.  (CEVDd : 0 - CEVD not diagnosed, 1 - CEVD diagnosed) | "脑栓塞" or "脑梗" or "硬膜?出血" or "蛛网膜出血" or "脑卒中" or " 脑血管畸形" or "脑血管瘤" or "脑出血" or " 颅内出血" |
| **Dementia (Dem)：** |  |
| DeMd (DEM diagnosed at admission/discharge)  DeMd = 0 if not = 1;  DeMd = 1 when Term are found in admission or discharge diagnoses.  (DeMd : 0 - DeM not diagnosed, 1 - DeM diagnosed) | "痴呆" |
| **Chronic Obstructive Pulmonary Disease (COPD)：** |  |
| COPDd (COPD diagnosed at admission/discharge)  COPDd = 0 if not = 1;  COPDd = 1 when Term are found in admission or discharge diagnoses.  (COPDd : 0 - COPD not diagnosed, 1 - COPD diagnosed) | "慢性阻塞性肺病" or "慢性梗阻性肺病" or "慢阻肺" or "肺气肿" or "老慢支" or "慢性支气管炎" |
| **Rheumatic Disease (RhD)：** |  |
| RhDd (RhD diagnosed at admission/discharge)  RhDd = 0 if not = 1;  RhDd = 1 when Term are found in admission or discharge diagnoses.  (RhDd : 0 - RhD not diagnosed, 1 - RhD diagnosed) | "风湿性" |
| **Peptic Ulcer Disease (PUD)：** |  |
| PUDd (PUD diagnosed at admission/discharge)  PUDd = 0 if not = 1;  PUDd = 1 when Term are found in admission or discharge diagnoses.  (PUDd : 0 - PUD not diagnosed, 1 - PUD diagnosed) | "消化性溃疡" or "胃&溃疡" or "十二指肠&溃疡" or "食管&溃疡" |
| **Mild Liver Disease (mLD) and Moderated to Severe Liver Disease (sLD)：** | |
| mLDd (mLD diagnosed at admission/discharge)  mLDd = 0 if not = 1 or 2;  mLDd = 1 when Term I are found in admission or discharge diagnoses;  mLDd = 2 when mLDd = 1 and Term II are found, or when Term III are found in admission or discharge diagnoses.  (mLD: 0 - mLD not diagnosed; 1 - mLD diagnosed; 2 - moderated to severe liver disease (**sLD**)) | I. "肝硬化" or "慢性乙" or "慢性丙" or "肝炎"  II. "失代偿" or "消化道" or "食道" or "腹水" or "肝性脑病" or "脾" or "肝昏迷" or "肝衰" or "咳血" or "呕血" or "门静脉" or "门脉"  III. "肝?衰" |
| **Diabetes Mellitus (DM) and Diabetes Mellitus with complications (DMc):** | |
| DMd (DM diagnosed at admission/discharge)  Searching Admission or Discharge Diagnoses  DMd = 0 if not = 1 or 2;  DMd = 1 when Term I are found and Term III is not found;  DMd = 2 when DMd = 1 and Term II are found.  (DMd: 0 - DM not diagnosed; 1 - DM diagnosed; 2 - DM with  complications (DMc))  DMs (DM blood sugar level )  DMs = 0 if not 1;  DMs = 1 when the result of Term IV >=7.0umol/l at least twice during the hospitalization. | I"糖尿病"  II. "肾" or "视网膜" or "足" or "神经病变"  III. "妊娠" or "孕"  IV. "空腹血糖" or "血糖" or "快速血糖" |
| Combination:  DM = 0 when DMd = 0 and DMs = 0,  DM = 1 when DMd = 1 or DMs = 1.  DM = 2 when DMd = 2.  (DM: 0 - DM not diagnosed; 1 - DM without complications diagnosed; 2 - DM with complications **(DMc)**) | |
| **Hemiplegia/ Paraplegia (HePa):** |  |
| HePad (He or Pa diagnosed at admission or discharge)  Searching Admission or Discharge Diagnoses  HePad = 0 if not = 1; HePad = 1 when Term are found (HePad: 0 - HePa not diagnosed; 1 - HePa diagnosed) | "偏瘫" or "截瘫" |
| **Moderate to severe Renal Disease (ReD):** |  |
| ReDd (ReD diagnosed at admission or discharge)  Searching Admission or Discharge Diagnoses  ReDd = 0 if not = 1; ReDd = 1 when Term are found (ReDd: 0 - ReD not diagnosed; 1 - ReD diagnosed) | "肾病" or "肾功能不全" or "肾功能损伤" or "肾衰" or "肾功能衰" or "肾功能衰竭" or "肾炎" or "肾结石" or "肾积水" or "肾盂炎" |
| **Any Malignance including Lymphoma and Leukemia (AM)：** |  |
| AMd (AM diagnosed at admission or discharge)  Searching Admission or Discharge Diagnoses  AMd = 0 if not = 1; AMd = 1 when Term I are found; AMd = 2 when AMd = 1 and Term II are found; AMd = 3 when AMd = 2 and Term III is not found.  (AMd: 0 - AM not diagnosed; 1 - AM diagnosed; 2 - AM with foundastasis; 3 - Metastatic Solid Tumor (MST)) | I. "恶性" or "淋巴瘤" or "肉瘤" or "白血病" or "恶性粒细胞" or "恶性淋巴细胞" or "恶性血细胞" or "骨髓瘤" or "胶质瘤" or "母细胞瘤" or "癌"  II. "转移"  III. "白血病" or "恶性粒细胞" or "恶性淋巴细胞" or "恶性血细胞" or "骨髓瘤" |
| **Acquired Immune Deficiency Syndrome (AIDS)：** |  |
| AIDSd (AIDS diagnosed at admission or discharge)  Searching Admission or Discharge Diagnoses  AIDSd = 0 if not = 1; AIDSd = 1 when Term are found (AIDSd: 0 - AIDS not diagnosed; 1 - AIDS diagnosed) | "艾滋" or "免疫缺陷综合" |
| **TREATMENTS/PROCEDURES** | |
| **Liver Transplantation (LT)：** |  |
| Searching the Operation/procedure records and the discharge summary.  LT = 0 if not = 1; LT = 1 when Term are found.  (LT: 0 - no LT performed; 1 - LT performed) | "同种异体原位肝移植术" or "原位肝移植" or "肝脏移植" or "同种异体肝移植术" or "原位肝移植术" or "肝脏移植术" or "异体肝移植术" or "原位肝移植" |
| **Hepatectomy (HT)：** |  |
| Searching the Operation/procedure records and the discharge summary.  HT = 0 if not = 1; HT = 1 when Term are found.  (HT: 0 - no HT performed; 1 - HT performed) | "肝癌切除术" or "肝癌切除" or "肝肿瘤切除术" or "叶切除" or "肝肿瘤剔除" or "半肝切除" or "肝脏肿瘤切除术" |
| **Esophagogastroduodenoscopy (EGD):** |  |
| Searching the Operation/procedure records and the discharge summary.  HT = 0 if not = 1; HT = 1 when Term are found.  (HT: 0 - no HT performed; 1 - HT performed) | "胃镜" or "食管胃十二指肠镜" or "胃十二指肠镜" |
| **Transhepatic Arterial Chemoembolization (TACE)：** | |
| Searching the Operation/procedure records and the discharge summary.  TACE = 0 if not = 1; TACE = 1 when Term I are found and Term II is not found; TACE = 2 when TACE = 1 and Term II are found.  (TACE: 0 - no TACE performed; 1 - TACE performed; 2 - Hepatic artery angiography performed) | I. "肝动脉" or "TACE"  II. "肝动脉导管造影" or "肝动脉造影 |
| **Radiation therapy (RT)：** |  |
| Searching the Operation/procedure records and the discharge summary.  RT = 0 if not = 1; RT = 1 when Term are found.  (RT: 0 - no RT performed; 1 - RT performed) | "放射" or "放疗" |
| [**Tumor Ablation**](http://www.cancer.org/ssLINK/liver-cancer-treating-tumor-ablation) **(TA):** |  |
| Searching the Operation/procedure records and the discharge summary.  TA = 0 if not = 1; TA = 1 when Term are found.  (TA: 0 - no TA performed; 1 - TA performed) | "射频" or "消融" |
| **Paracentesis (Pa):** |  |
| Searching the Operation/procedure records and the discharge summary.  Pa = 0 if not = 1; Pa = 1 when Term are found.  (Pa: 0 - no Pa performed; 1 - Pa performed) | "腹穿" or "腹腔穿" |
| **Chinese Herb Medication (CHM):** |  |
| Searching the discharge summary.  CHM = 0 if not = 1; CHM = 1 when Term are found.  (CHM: 0 - not visit to Chinese Medication Department; 1 - Chinese Medication Department) | "中医" |
| **ELIXAUSER COMORBIDITIES** | |
| **Cardiac Arrhythmias (CA):** |  |
| CAad/dis (CA diagnosed at admission or discharge)  Searching Admission or Discharge Diagnoses  CAad/dis = 0 if not = 1; CAad/dis = 1 when Term are found  (CAad/dis: 0 - CA not diagnosed; 1 - CA diagnosed) | "心率失" or "心率不齐" or "房颤" or "室颤" or "心律失" or "心律不齐" |
| **Valvular Disease (VD):** |  |
| VDad/dis (VD diagnosed at admission or discharge)  Searching Admission or Discharge Diagnoses  VDad/dis = 0 if not = 1 or 2; VDad/dis = 1 when Term I are found; VDad/dis = 2 when VDad/dis = 1 and Term II are found.  (VDad/dis: 0 - VD not diagnosed; 1 - VD diagnosed; 2 - patient with VD and underwent related operation) | I. "瓣膜性心脏病" or "二尖瓣" or "心脏瓣膜病" or "瓣膜病" or "主动脉瓣关闭不全"  II. "术后" |
| **Hypertension, complicated (Hpc) and Hypertension without complications (Hpu):** | |
| Hpcad/dis (Hpc diagnosed at admission or discharge)  Searching Admission or Discharge Diagnoses  Hpcad/dis = 0 if not = 1 or 2; Hpcad/dis = 1 when Term I are found and Term II are found but Term III is not found; Hpcad/dis = 2 when only Term I are found.  (Hpcad/dis: 0 - Hpc not diagnosed; 1 - Hpc diagnosed; 2 - hypertension without complications (**Hpu**)) | I. "高血压"  II. "肾病" or "左心室肥厚" or "心绞痛" or "脑病" or "硬化" or "视网膜" or "眼病" or "高危" or "极高危"  III. "妊娠" or "孕" |
| **Hypothyroidism (Hy):** |  |
| Hyad/dis (Hy diagnosed at admission or discharge)  Searching Admission or Discharge Diagnoses  Hyad/dis = 0 if not = 1; Hyad/dis = 1 when Term are found.  (Hyad/dis: 0 - Hy not diagnosed; 1 - Hy diagnosed) | "甲状腺功能减退" or "甲减" |
| **Lymphoma (Ly):** |  |
| Lyad/dis (Ly diagnosed at admission or discharge)  Searching Admission or Discharge Diagnoses  Lyad/dis = 0 if not = 1; Lyad/dis = 1 when Term are found.  (Lyad/dis: 0 - Ly not diagnosed; 1 - Ly diagnosed) | "淋巴瘤" or "淋巴细胞瘤" |
| **Coagulopathy (Cop):** |  |
| Copad/dis (Cop diagnosed at admission or discharge)  Searching Admission or Discharge Diagnoses  Copad/dis = 0 if not = 1; Copad/dis = 1 when Term are found.  (Copad/dis: 0 - Cop not diagnosed; 1 - Cop diagnosed) | "凝血功能障碍" or "凝血障碍" |
| **Anemia:** |  |
| Anemiaad/dis (Anemia diagnosed at admission or discharge)  Searching Admission or Discharge Diagnoses  Anemiaad/dis = 0 if not = 1; Anemiaad/dis = 1 when Term I are found and Term IV is not found; Anemiaad/dis = 2 when Anemiaad/dis = 1 and Term II are found; Anemiaad/dis = 3 when Anemiaad/dis = 1 and Term III are found.  (Anemiaad/dis: 0 - Anemia not diagnosed; 1 - Anemia diagnosed; 2 - Blood Loss Anemia (**BLA**); 3 - Deficiency Anemia (**DeA**)) | I. "贫血"  II. "失血"  III. "缺铁"  IV. "肾性贫血" |
| **Depression (Dep)：** |  |
| Depad/dis (Dep diagnosed at admission or discharge)  Searching Admission or Discharge Diagnoses  Depad/dis = 0 if not = 1; Depad/dis = 1 when Term are found.  (Depad/dis: 0 - Dep not diagnosed; 1 - Dep diagnosed) | "抑郁" |
| **Fluid and Electrolyte Disorders (FED)：** |  |
| FEDad/dis (FED diagnosed at admission or discharge)  Searching Admission or Discharge Diagnoses  FEDad/dis = 0 if not = 1; FEDad/dis = 1 when Term are found.  (FEDad/dis: 0 - FED not diagnosed; 1 - FED diagnosed) | "水电解质紊乱" or "水电解质离子紊乱" or "电解质紊乱" or "离子紊乱" |
| **Weight Loss (WL)：** |  |
| WLad/dis (WL diagnosed at admission or discharge)  Searching Admission or Discharge Diagnoses  WLad/dis = 0 if not = 1; WLad/dis = 1 when Term are found.  (WLad/dis: 0 - WL not diagnosed; 1 - WL diagnosed) | "体重减低" or "体重降低" |
| **Psychoses (Ps)：** |  |
| Psad/dis (Ps diagnosed at admission or discharge)  Searching Admission or Discharge Diagnoses  Psad/dis = 0 if not = 1; Psad/dis = 1 when Term are found.  (Psad/dis: 0 - Ps not diagnosed; 1 - Ps diagnosed) | "精神病" or "精神分裂" or "强迫" or "躁狂" |
| **Renal Failure (RF):** |  |
| RFad/dis (RF diagnosed at admission or discharge)  Searching Admission or Discharge Diagnoses  RFad/dis = 0 if not = 1; RFad/dis = 1 when Term are found.  (RFad/dis: 0 - RF not diagnosed; 1 - RF diagnosed) | "肾功能不全" or "肾功能损伤" or "肾衰" or "肾功能衰" or "肾功能衰竭" |
| **Solid tumor without metastasis (ST):** |  |
| STad/dis (ST diagnosed at admission or discharge)  Searching Admission or Discharge Diagnoses  STad/dis = 0 if not = 1; STad/dis = 1 when Term are found.  (STad/dis: 0 - ST not diagnosed; 1 - ST diagnosed) | "恶性乳腺" or "恶性黑色素瘤" or "胃癌" or "肠癌" or "肺癌" or "肾癌" or "淋巴瘤" or "肉瘤" or "骨髓瘤" or "胶质瘤" or "母细胞瘤" |

Notes:

1. Chronic conditions (e.g., PLC) were diagnosed at discharge or during the hospitalization will be considered present at admission given that chronic diseases have very low possibility of developing during the hospitalization.
2. Laboratory test or radiology result at admission is the one nearest to the admission date, similarly, the laboratory test or radiology result at discharge is the one nearest to the discharge date.
3. The term ("word 1" & "word 2") means we aimed to find the targets containing "word 1" and "word 2" at the same time no matter what existed prior/posterior/between the two words. Similarly, the term ("word 1" & "word 2" & "word 3") means we aimed to find the targets containing the 3 words at the same time no matter how their order is.
4. The sign "?" in the search term represents unlimited words or spaces.

**Table 2 Validity results of the 29 EMR definitions for comorbidities and t**reatments

| **Variable** | **Percent**  **%** | **Sensitivity**  **%, (95% CI*)** | **Specificity**  **% (95% CI)** | **PPV****  **% (95% CI)** | **NPV*****  **% (95% CI)** |
| --- | --- | --- | --- | --- | --- |
| **CHARLSON COMORBIDITES (CC)** | | | | | |
| **Myocardial Infarction (MI)** | 0.4 | 100.0  (15.8, 100.0) | 99.8  (98.7, 100.0) | 66.7  (9.4, 99.2) | 100.0  (99.2, 100.0) |
| **Cerebrovascular Disease (CEVD)** | 1.8 | 100.0  (63.1, 100.0) | 99.8  (98.8, 100.0) | 88.9  (51.8, 99.7) | 100.0  (99.2, 100.0) |
| **Chronic Obstructive Pulmonary Disease (COPD)** | 1.1 | 100.0  (47.8, 100.0) | 100.0  (99.2, 100.0) | 100.0  (47.8, 100.0) | 100.0  (99.2, 100.0) |
| **Rheumatic Disease (RhD)** | 0.2 | 100.0  (2.50, 100.0) | 100.0  (99.2, 100.0) | 100.0  (2.5, 100.0) | 100.0  (99.2, 100.0) |
| **Peptic Ulcer Disease (PUD)** | 3.8 | 64.7  (38.3, 85.8) | 99.8  (98.7, 100.0) | 91.7  (61.5, 99.8) | 98.6  (97.0, 99.5) |
| **Diabetes Mellitus with complications (DMc)** | 0.7 | 100.0  (29.2, 100.0) | 99.8  (98.8, 100.0) | 75.0  (19.4, 99.4) | 100.0  (99.2, 100.0) |
| **Diabetes Mellitus (DM)** | 19.1 | 100.0  (95.8, 100.0) | 98.6  (96.8, 99.6) | 94.5  (87.6, 98.2) | 100.0  (98.9, 100.0) |
| **Renal Disease (ReD)** | 5.8 | 96.2  (80.4, 99.9) | 100. 0  (99.1, 100.0) | 100.0  (86.3, 100.0) | 99.7  (98.7, 100.0) |
| **Any Malignance (AM)** | 21.3 | 99.0  (94.3, 100.0) | 93.8  (90.7, 96.0) | 81.2  (72.9, 87.8) | 99.7  (98.3, 100.0) |
| **Metastatic Solid Tumor (MST)** | 2.4 | 72.7  (39.0, 94.0) | 99.5  (98.4, 99.9) | 80.0  (44.4, 97.5) | 99.3  (98.0, 99.9) |
| **Acquired Immune Deficiency Syndrome (AIDS)** | 2.9 | 100.0  (75.3, 100.0) | 100.0  (99.2, 100.0) | 100.0  (75.3, 100.0) | 100.0  (99.2, 100.0) |
| **ELIXHAUSER COMORBIDITIES (Excluded conditions included in CC)** | | | | | |
| **Cardiac Arrhythmias (CA)** | 1.6 | 100.0  (59.0, 100.0) | 99.8  (98.8, 100.0) | 87.5  (47.4, 99.7) | 100.0  (99.2, 100.0) |
| **Hypertension (Hp)** | 12.2 | 100.0  (93.5, 100.0) | 99.8  (98.6, 100.0) | 98.2  (90.5, 100.0) | 100.0  (99.1, 100.0) |
| **Hypertension without complications (Hpu)** | 7.3 | 75.8  (57.7, 88.9) | 99.8  (98.7, 100.0) | 96.2  (80.4, 99.9) | 98.1  (96.3, 99.2) |
| **Hypertension with complications (Hpc)** | 5.3 | 95.8  (78.9, 99.9) | 98.1  (96.3, 99.2) | 74.2  (55.4, 88.1) | 99.8  (98.7, 100.0) |
| **Valvular Disease (VD)** | 0.7 | 66.7  (9.4, 99.2) | 100.0  (99.2, 100.0) | 100.0  (15.8, 100.0) | 99.8  (98.7, 100.0) |
| **Hypothyroidism (Hy)** | 0.4 | 100.0  (15.8, 100.0) | 100.0  (99.2, 100.0) | 100.0  (15.8, 100.0) | 100.0  (99.2, 100.0) |
| **Anemia** | 10.0 | 100.0  (92.1, 100.0) | 99.8  (98.6, 100.0) | 97.8  (88.5, 99.9) | 100.0  (99.1, 100.0) |
| **Depression (Dep)** | 0.2 | 100.0  (2.5, 100.0) | 100.0  (99.2, 100.0) | 100.0  (2.5, 100.0) | 100.0  (99.2, 100.0) |
| **Fluid and Electrolyte Disorders (FED)** | 5.8 | 65.4  (44.3, 82.8) | 100.0  (99.1, 100.0) | 100.0  (80.5, 100.0) | 97.9  (96.1, 99.1) |
| **Renal Failure (RF)** | 1.3 | 100.0  (54.1, 100.0) | 100.0  (99.2, 100.0) | 100.0  (54.1, 100.0) | 100.0  (99.2, 100.0) |
| **Solid Tumor without metastasis (ST)** | 20.7 | 98.9  (94.2, 100.0) | 93.0  (89.8, 95.4) | 78.6  (70.1, 85.7) | 99.7  (98.3, 100.0) |
| **TREATMENTS/PROCEDURES** | | | | | |
| **Liver Transplantation (LT)** | 1.3 | 100.0  (54.1, 100.0) | 100.0  (99.2, 100.0) | 100.0  (54.1, 100.0) | 100.0  (99.2, 100.0) |
| **Hepatectomy (HT)** | 0.2 | 100.0  (2.5, 100.0) | 100.0  (99.2, 100.0) | 100.0  (2.5, 100.0) | 100.0  (99.2, 100.0) |
| **Transcatheter Arterial Chemoembolization (TACE)** | 4.4 | 100.0  (83.2, 100.0) | 98.8  (97.3, 99.6) | 80.0  (59.3, 93.2) | 100.0  (99.1, 100.0) |
| **Esophagogastroduodenoscopy (Egd)** | 11.8 | 77.4  (63.8, 87.7) | 94.2  (91.4, 96.3) | 64.1  (51.1, 75.7) | 96.9  (94.6, 98.4) |
| **Tumor Ablation (TA)** | 1.3 | 100.0  (54.1, 100.0) | 99.8  (98.8, 100.0) | 85.7  (42.1, 99.6) | 100.0  (99.2, 100.0) |
| **Paracentesis (Pt)** | 6.4 | 100.0  (88.1, 100.0) | 99.5  (98.3, 99.9) | 93.6  (78.6, 99.2) | 100.0  (99.1, 100.0) |
| **Chinese Herb Medication (CHM)** | 3.8 | 100.0  (80.5, 100.0) | 100.0  (99.2, 100.0) | 100.0  (80.5, 100.0) | 100.0  (99.2, 100.0) |

CI: confidence interval; PPV: positive predictive value; NPV: negative predictive value
